# Carrier priming to improve pneumococcal disease control and reduce the international program’s cost in children

**DOI:** 10.1186/s41479-016-0016-8

**Published:** 2016-09-27

**Authors:** Mohamed Tashani, Harunor Rashid, Kim Mulholland, Robert Booy

**Affiliations:** 1 National Centre for Immunisation Research and Surveillance (NCIRS), The Children’s Hospital at Westmead, Sydney, NSW Australia; 2 The Discipline of Child and Adolescent Health, Sydney Medical School, University of Sydney, Sydney, NSW Australia; 3 NHMRC Centre for Research Excellence-Immunisation in understudied and special risk populations: closing the gap in knowledge through a multidisciplinary approach, School of Public Health and Community Medicine, Faculty of Medicine, University of New South Wales, Sydney, Australia; 4 Marie Bashir Institute for Infectious Diseases and Biosecurity, School of Biological Sciences and Sydney Medical School, University of Sydney, Sydney, Australia; 5 Murdoch Childrens Research Institute, Melbourne, VIC Australia; 6 London School of Hygiene and Tropical Medicine, London, UK; 7 WHO Collaborating Centre for Mass Gatherings and High Consequence/High Visibility Events, Flinders University, Adelaide, 5001 Australia

**Keywords:** Pneumococcal conjugate vaccine, Carrier priming

## Abstract

Pneumococcal conjugate vaccine (PCV) has the potential to interact with other vaccines containing diphtheria toxin-like antigens (such as those found in the DTP vaccine) upon sequential administration. This is attributed to the similarity of the diphtheria toxoid antigen to the carrier protein used to make PCV, (known as cross reactive material [CRM]) to diphtheria toxin _197_ or CRM_197_. The interaction could lead to enhanced immunogenicity of PCV as a result of a phenomenon called carrier priming, whereby DTP is given some weeks before the first dose of PCV. This phenomenon could be implemented in the immunisation schedule in developing countries and among vulnerable populations to enhance the immunogenicity of PCV, reduce the number of doses required, and produce a more cost-effective immunisation program in developing countries.

## Main text

*Streptococcus pneumoniae* is a leading cause of pneumonia and death in children worldwide [1]. It is estimated that 14.5 million episodes of serious pneumococcal disease occur each year in children aged less than five years, resulting in at least 500,00 deaths, almost all of which occur in low- and middle-income countries [2]. Vaccination has proved very successful in the control of pneumococcal disease in many developing countries and there is growing evidence for herd protection. For example, in The Gambia, infant vaccination has reduced vaccine-type pneumococcal carriage not only among vaccinated children but also among non-vaccinated older children and adults, indicating a substantial herd effect [3]. However, not all countries have introduced vaccination of infants as implementation is limited by the high cost of the vaccine [4]. The cost of one dose of pneumococcal conjugate vaccine (PCV) can be very high in developed countries (> $100) [5]. The best subsidised price for resource-poor countries has been brought down to $3 · 30 per dose [6], which is comparable to the cost of all other pediatric vaccines combined (HepB, BCG, OPV, DPT-Hib, and Measles) [7]. Therefore, there is intense interest in reducing the cost of vaccination against pneumococcal disease in young children through eg. shorter schedules of one or two doses instead of three doses [8].

The use of the 3 + 0 schedule is supported by randomised controlled clinical trials for prevention of pneumonia and invasive pneumococcal disease (IPD) in developing countries [9], and has been recommended by WHO. However, after more than a decade of using PCV in many developed countries, evidence consistently demonstrates strong herd immunity; most vaccine-type pneumococcal disease is close to elimination in children irrespective of individual vaccination status [10]. Consequently, a “1 + 1” schedule (where a booster dose is given in second year of life) has been promoted for use in developing countries where the immunisation program is mature (implemented for few years) and vaccine-type carriage is largely eliminated in the community [11]. In such mature vaccine programmes, individual protection may not be required because the probability of exposure to vaccine-type infection has become very low [10]. Therefore, a “1 + 1” schedule could be sufficient to maintain disease control, and at a considerably reduced cost [8]. Long-term protection against IPD by PCV depends on a combination of persistence of protective serum antibody levels, immunological memory, and herd immunity [12]. Additionally, and importantly, we suggest consideration of the use of carrier priming to enhance the immunogenicity, especially of the first PCV dose. Carrier priming is defined as an improved antibody response to a carbohydrate portion of a glycoconjugate vaccine because an individual has been previously primed with the carrier protein [13].

The 13-valent PCV utilises carrier protein cross-reacting material 197 (CRM197) of diphtheria toxin, which is antigenically similar to the diphtheria toxin in DTP. It is believed that priming via the carrier molecule enhances the response to conjugate vaccines by increasing the number of carrier-specific T lymphocytes; these can provide the necessary support for the expansion and differentiation of polysaccharide specific B lymphocytes [14]. Several studies have found that prior receipt of tetanus/diphtheria containing vaccine such as DTP in infants who are then administered conjugate vaccine can lead to rapid and earlier onset of clinical protection against the disease [15-17]. In developing countries, the risk of IPD peaks in the first few months of life and the current vaccine schedule is 6, 10 and 14 weeks. We propose evaluation of a schedule that offers the first PCV at ten weeks of age after prior administration of DTP vaccine at six weeks to take advantage of carrier priming [16]. If adequate priming occurs, a reduced schedule (where first PCV is given after DTP) may be sufficient and more cost-effective, particularly for resource poor settings with mature immunisation programs.

## Abbreviations

BCG: Bacille Calmette Guerin vaccine
CRM197: Cross-reacting material 197
DPT-Hib: Diphtheria tetanus pertussis vaccine and haemophilus influenzae type b vaccine
HepB: Hepatitis B vaccine
OPV: Oral polio vaccine
IPV: Invasive pneumococcal disease
PCV: Pneumococcal conjugate vaccine
